# Current advances in the selection of adjuvant radiotherapy regimens for keloid

**DOI:** 10.3389/fmed.2022.1043840

**Published:** 2022-11-08

**Authors:** Weihao Wang, Jiang Zhao, Chi Zhang, Wanqi Zhang, Manqiu Jin, Ying Shao

**Affiliations:** ^1^Department of Plastic and Reconstructive Surgery, The First Hospital of Jilin University, Changchun, China; ^2^Department of Urology, The First Hospital of Jilin University, Changchun, China

**Keywords:** keloid, adjuvant radiotherapy, radiation, dose, recurrence

## Abstract

Keloid is a common benign skin tumor in the outpatient department, and patients are often accompanied by itching and pain. Since the pathogenesis is unknown, the effect of single method treatment is unsatisfactory, and therefore the recurrence rate is high. Therefore, comprehensive treatment is mostly used in clinical treatment. Adjuvant radiotherapy is currently one of the most effective treatments for keloid. After long-term clinical practice, brachytherapy and electron beam radiotherapy has increasingly become the gold standard of treatment, because brachytherapy provides more focused radiation treatment to focal tissue to significantly reduce recurrence rate, and better preserve normal tissue. With the development of new radiotherapy techniques, more options for the treatment of keloid. Currently, adjuvant radiotherapy has been widely recognized, but there is no consensus on the optimal protocol for adjuvant radiotherapy for keloids. This review provides a review of published treatment options and new radiotherapy techniques for adjuvant radiotherapy of keloids and gives a comprehensive evaluation for clinical treatment.

## Introduction

Keloid is a dermatofibrosis characterized by excessive fibroblast proliferation and collagen deposition that invades normal tissue beyond its original borders, often secondary to trauma, surgery, and inflammation (1, 2), with an incidence of 5–15% during wound healing (3). Keloid often develops in patients with a family history of the disease or dark-skinned patients (4, 5). Due to the high recurrence rate, this disease remains one of the most pressing clinical challenges. The recurrence rate of keloids in patients who received adjuvant radiation therapy has been reported to be approximately 20%, while the recurrence rate in patients who underwent surgery alone ranged from 50 to 99% (6–8). In addition, Mankowski et al. (9) showed that patients receiving adjuvant radiotherapy also had a lower recurrence rate than those receiving radiation therapy alone (22 and 37%). Therefore, postoperative adjuvant radiotherapy for keloids significantly reduces the recurrence rate (10). Radiotherapy uses radiation to deliver energy to cells to reduce recurrence rates by causing changes in fibroblast structure and activity through direct and indirect effects, thereby killing cells to reduce overproliferation (11). Radiation itself has some carcinogenic potential and therefore the surrounding tissues should be provided with the necessary protection during treatment, especially in children (12, 13). A review of the literature detailed that radiotherapy for keloids induces cancer including fibrosarcoma, basal cell carcinoma, thyroid cancer, and breast cancer, but the probability is extremely low (14). Although radiotherapy has the potential to induce tumors, the risk of surrounding tissue carcinoma from the dose and frequency of radiation used to treat keloids is very low (15, 16). And a survey showed that 78% of oncologic radiologists considered radiotherapy an acceptable treatment for keloids (17). Radiotherapy modalities used to treat keloids include x-ray therapy, electron beam therapy, and brachytherapy (18, 19). This review summarizes the progress of research on different radiation modalities and treatment options in postoperative adjuvant radiotherapy for keloids.

## Methodology

In this review, we conducted a literature search for studies related to keloid scars using PubMed, Web of Science, Embase, and Cochrane. The keywords used in the search were “keloid,” “adjuvant radiotherapy,” and “treatment protocol.” Review articles were used as an initial source of information and, where relevant, information was obtained from primary research papers.

## Mechanism of radiotherapy

Radiation therapy has been used for the surgical or non-surgical treatment of keloids (8). Currently, the mechanism of radiation therapy for keloids is unclear, but researchers have provided multiple theoretical bases. At the cellular level, postoperative incisions are dominated by naive fibroblasts and unstable collagen fibers, which are more sensitive to radiation, and their damage by ionization can lead to impairment of cell migration, proliferation, and synthetic-secretory functions, thus affecting fibroblast proliferation and collagen synthesis, blocking the cell cycle and inducing apoptosis, and thus inhibiting keloid recurrence (13). A period of a few weeks with premature differentiation and senescence was observed after an immediate cell cycle arrest (20). And after cellular exposure to radiation, the levels of reactive oxygen species such as superoxide (O^2^-), hydrogen peroxide (H^2^O^2^), and hydroxyl radicals (-OH) increase dramatically, leading to damage to macromolecules and DNA (21–24), and thus inhibiting fibroblast proliferation.

However, at the molecular level, radiation inhibited fibroblast proliferation and induced cellular senescence (11). The mRNA and protein expression of senescence-associated genes p16, p21, and p27 were increased in a time-dependent manner after 4 Gy irradiation. This expression is followed by the activation of a dynamic feedback loop by p21, followed by mitochondrial dysfunction and elevated levels of reactive oxygen species, leading to DNA damage and sustained DNA damage reaction (25). Thus, the survival of fibroblasts after radiation-induced cell cycle arrest depends not only on persistent DNA damage and p21 levels but mainly on the cellular CDK2/p21 ratio (26). Tosa et al. (27) combined the results from primary fibroblasts isolated from keloids of five patients who were subjected to irradiation with 15 Gy of electrons, and after 15 min they were compared with unirradiated primary fibroblasts and normal fibroblasts, and the results showed that after irradiation, the expression of 28 genes of keloid fibroblasts was upregulated, accounting for 29.2% of the total number of genes; meanwhile, the expression of 68 genes was downregulated, accounting for the expression of 68 genes was down-regulated, accounting for 70.8% of the total number of genes. Among them, several down-regulated genes were involved in the regulation of cell proliferation and extracellular matrix production, while some up-regulated genes were involved in the regulation of apoptosis and extracellular matrix degradation. Ji et al. (11) found that radiotherapy dysregulated the proliferation and apoptosis of keloid fibroblasts and inhibited the proliferation of keloid fibroblasts compared to normal fibroblasts. Li et al. (28) found that miR-21 was highly expressed in keloid tissues, and its expression level decreased after 15 Gy electron irradiation. Synthesis and this process are affected by electron beam irradiation, therefore, electron beam irradiation can reduce the production of type I collagen by further activating p38 protein through miR-21/Smad7. This study explored the effect of electron beam irradiation on microRNA (miRNA) for the first time, which provides a new idea to discover the molecular mechanism of radiotherapy for keloid treatment. Yan et al. (29) showed that ionizing radiation can inhibit autophagy and promote apoptosis in keloid cells by reducing the expression level of miR-21-5p, and miR-21-5p can regulate this process through the human chromosome 10 deletion phosphatase and tensin homolog (PTEN) and phosphorylated protein kinase B (p-AKT) signaling pathways, thereby preventing local invasion and recurrence of keloids.

Dysregulated long noncoding RNAs (LncRNAs) play an important role in keloid formation by regulating many processes, including fibroblast proliferation and extracellular matrix deposition. Recent studies have shown that LncRNA also has an important role in the formation of keloids (30). The development of keloids is remarkably associated with fibroblast proliferation, invasion, migration, and apoptosis. LncRNA and miRNA can interact and co-regulate with each other. LncRNA is known as a competitive endogenous RNA and can negatively regulate miRNA to influence its activity (31). LncRNA can also prevent miRNA from acting on mRNA and can enhance the translation of mRNA. Over 2,500 LncRNAs were found to be differentially expressed between keloid tissue and normal human skin by microarray and qRT-PCR (32). Of these, 1,731 LncRNAs were upregulated and 782 LncRNAs were downregulated (33). In another study, a total of 2,068 LncRNAs were found to be differentially expressed in earlobe keloids by microarray. Of these, 1,290 LncRNAs were up-regulated and 778 LncRNAs were down-regulated (34). Among them, H19 promotes proliferation by targeting miR-29a and miR-214-5p in keloid fibroblasts (35–37). HOXA11-AS promotes proliferation and migration by targeting miR-124-3p and miR-205-5p in keloid fibroblasts (38–40). CAS1 promotes proliferation, invasion, and migration by targeting miR-205 in keloid fibroblasts and inhibits the apoptotic process (41, 42). LINC01116 promotes proliferation, invasion, and migration by targeting miR-203 and miR-3141 in keloid fibroblasts and produces an extracellular matrix, and inhibits the apoptotic process (43, 44). LncRNA-ATB was upregulated in keloid fibroblasts by targeting miR-200c and ZNF217 to promote their TGF-β2 secretion (45, 46). AC073257.2 was upregulated in keloid fibroblasts by targeting GLI2 to promote their growth and proliferation (47). LINC00937 was upregulated in keloid fibroblasts by targeting miR-28-5p down-regulation in keloid fibroblasts to inhibit their proliferation and extracellular matrix deposition (48). The efficacy of radiotherapy depends mainly on the sensitivity of the irradiated cells to radiotherapy. Some LncRNAs and miRNAs can enhance the sensitivity of cells to radiotherapy, mainly by regulating different genes involved in processes closely related to radiotherapy sensitivity, including affecting DNA damage and inducing cell cycle arrest, regulating the process of DNA damage repair and apoptosis, and activating EGFR signaling (49–56). As radiotherapy research advances, the role of LncRNA for keloid prognosis prediction, treatment process monitoring, and response prediction will certainly become clearer and has the potential to become a potential biomarker for keloids.

## Radiation therapy techniques

**FIGURE 1:**
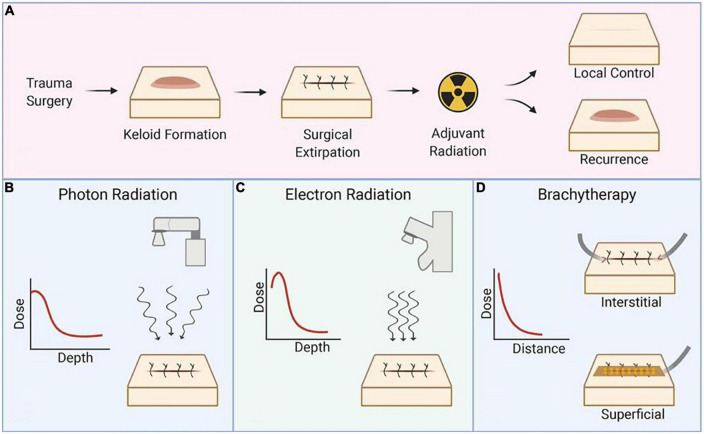
Schematic representation of the radiation technique. **(A)** Process and prognosis of postoperative radiotherapy for keloids, **(B)** mechanism of X-ray and selection of dose and depth, **(C)** mechanism of electron radiation and selection of dose and depth, and **(D)** mechanism of brachytherapy and selection of dose and depth. Reproduced with permission from Journal of Plastic, Reconstructive & Aesthetic Surgery (19).

Postoperative adjuvant radiotherapy is currently the most effective clinical treatment for keloids (10, 57, 58). Commonly used are external beam radiotherapy (EBRT) and brachytherapy, with EBRT being the earliest radiotherapy method used, consisting mainly of X-ray and electron beam (59). The recurrence rate of high dose rate (HDR) brachytherapy was significantly lower than ERBT, but the recurrence time of keloids was later with EBRT than with brachytherapy, with a mean delay of 2.5 years (60). The relationship between their modes of action, as well as dose and depth, is shown in Figure 1.

## X-ray

The X-ray can be produced by X-ray tubes and usually deliver superficial low-dose radiation to the lesion. In 1906, De Bearman and Gourgerot first used X-ray to treat keloids (61). In 1961, Cosman et al. (62) proposed that X-ray treatment be performed immediately after surgical excision of keloids to prevent a recurrence. Studies have shown a recurrence rate of only 21% and a controlled rate of 73–88% for immediate postoperative x-ray treatment (63, 64). Son et al. (65) treated 20 keloids in 15 patients with a single dose of 8 Gy at 50 kV of X-ray and followed up for 6 months, showing a recurrence rate of 6.25%. As revealed in Table 1, the shorter treatment time and the lower dose required for X-ray compared to other radiotherapy modalities resulted in a lower incidence of acute skin complications. However, there are some defects in the treatment of keloids, such as the greater penetration of X-ray and the greater damage to the surrounding normal tissues; the lack of strict radiotherapy protocols, definite histopathological results, and clear consensus on complications; and the lack of follow-up time and the bias of statistical results due to the assessment of efficacy by recurrence rate only, which is rarely used in the treatment of keloids nowadays. Most reports on x-ray are limited to small samples in single-center retrospective studies. The recurrence rates in this literature are within the range of outcomes typically reported for EBRT (6). Despite the low frequency of clinical application of x-ray, the available literature supports its value as an equally effective and safe form of radiotherapy. It may be used when the extent and complexity of the keloid lesion are such that excessive consideration of damage to the surrounding tissue is not allowed.

**TABLE 1:** Dose fractionation and recurrence rate of x-ray for keloids.

Case (keloids)	Total dose/Fraction	Interval (hour)	Recurrence rate	References
(68)	20 Gy/4	14 days	23.5%	(103)
(28)	16 Gy/4	14 days	21.%	
14 (15)	(8–10) Gy/2	24	–	(104)
1	15 Gy/3	2	–	(105)
84 (197)	(9–30) Gy/(1–10)	36	19%	(60)
(188)	16 Gy/4; 24 Gy/6; 30 Gy/10	168	33%	(106)
64 (86)	37.5 Gy/5	–	–	(107)

## Electron beam

With advances in radiation technology, X-ray is gradually being replaced by accelerator-generated electron beams. Although some studies have shown that the recurrence rate of postoperative electron beam treatment of keloids is not significantly different from that of x-ray treatment (9, 66). However, the energy is delivered to a depth of 2–5 cm by the accelerator to keloids, and electron beam radiotherapy is more suitable than x-ray therapy for flat and lumpy keloids (67). Compared with X-ray, the more concentrated radiation coverage is more suitable for treating localized superficial lesions such as keloids without causing serious damage to deeper tissues while treating them. Electron beam treatment of keloids often uses energies of 4–6 MeV, and electron beam treatment after surgical excision of keloids can significantly reduce the recurrence rate, and its effectiveness is closely related to the treatment protocol and the site of the keloid (68). Ogawa et al. (69) showed that the local recurrence rate was 5.7% for keloids in the earlobe, 14.3–20% in the chest, deltoid region, and suprapubic region, and 16.7% in other areas. Wang et al. (70) performed electron beam therapy in 58 patients within 4 h after surgery, and the treatment regimen was 12 Gy total dose completed in 4 sessions for keloids in the earlobe and neck, and 16 Gy total dose completed in 4 sessions for keloids in other sites; all patients recovered well with a recurrence rate of 8.6% at 22 months of follow-up. Thus, the choice of electron beam radiotherapy dose depends on the site of the keloid, with keloids in high tension areas (chest, scapular, and suprapubic areas) requiring higher doses for treatment compared to keloids in low tension areas (neck and auricle). As revealed in Table 2, the electron beam therapy produced by accelerators has a stable, controlled, and safe irradiation dose. Electron beam radiotherapy is one of the most common radiation modalities for keloid, and is often chosen when the scar is large and tends to be flat. Currently there are different treatment protocols depending on the site, with 18 Gy in 3 sessions for high recurrence sites, 8 Gy in 1 session for low recurrence sites, and 15 Gy in 2 sessions for other body parts. Keloids on the earlobe are usually adequately controlled with a single session, while keloids on high recurrence areas such as the anterior chest wall are treated with multiple and higher cumulative doses (71).

**TABLE 2:** Dose fractionation and recurrence rate of electron beam for keloids.

Case (keloids)	Total dose/Fraction	Interval (hour)	Recurrence rate	References
95	(9–32) Gy/(3–4)	24–48	–	(108)
11 (20)	13.5 Gy/3	24	–	(109)
58	(12–16) Gy/4	4	8.6%	(70)
14	12 Gy/3	24	–	(104)
23 (30)	15 Gy/3	2	–	(110)
30 (47)	(15–20) Gy/(3–4)	24 (36.7%); 14 days (63.3%)	–	(111)
9	15 Gy/3	2	6%	(105)
30 (37)	(5–12) Gy/(1–3)	24–48	16.2%	(112)
124 (250)	20 Gy/5; (12–16) Gy/(3–4)	48	1.6%(20 Gy); 9.6%(< 20 Gy)	(77)
568 (834)	18 Gy/2	48 (82.7%); > 48 (17.3%)	9.59%	(86)
30 (37)	(12–18) Gy/(3–6)	24 (64.9%); 24–72 (16.2%); 72 (18.9%)	18.9%	(91)
45 (45)	(15–20) Gy/(3–4)	24	2.2%	(113)
12 (16)	10 Gy/1	72	0	(114)
60 (91)	20 Gy/5; 16 Gy/4	72	50.5%	(115)
119 (194)	16 Gy/4; 24 Gy/6; 30 Gy/10	168	33%	(106)
96 (102)	15 Gy/3	24	45.8%	(116)
218 (249)	15 Gy/3	48	29.3%	
109 (121)	(10–20) Gy/(2–4)	48	14%	(69)
47 (60)	16 Gy/4	24 (83%); 24 (17%)	15%	(117)
64 (86)	37.5 Gy/5	–	–	(107)
129 (147)	15 Gy/3	–	32.7%	(118)
100 (134)	(5–18) Gy/(2–6)	24 (86%); 48 (14%)	16%	(119)

## Brachytherapy

Brachytherapy was first used for the treatment of keloids in 1967 (72), because high-dose ERBT may damage the normal tissue surrounding the lesion. There was no significant trend in local control with brachytherapy compared to electron beam radiotherapy (86.4% for HDR and 85% for EBRT) (73). However, compared with X-ray or electron beam, brachytherapy significantly reduces the recurrence of keloid (9, 10, 74). Brachytherapy is more effective in postoperative keloids. This therapy allows the radiation source to be placed at the target site, providing more focused radiation therapy to the focal tissue and better preservation of normal tissue (75). Brachytherapy can be divided into interstitial brachytherapy and surface brachytherapy. As shown in Table 3, the overall recurrence rate of HDR brachytherapy was low.

**TABLE 3:** Dose fractionation and recurrence rate of brachytherapy for keloids.

	Case (keloids)	Total dose/Fraction	Interval (hour)	Recurrence rate	References
HDR	31 (42)	(15–20) Gy/(3–4)	3 days; 4 days	44.4%	(120)
	50 (71)	15 Gy	24	2%	(121)
	14 (14)	12 Gy/3	24	21%	(122)
	13 (20)	8 Gy/1	36	–	(123)
	43 (86)	18 Gy/2	–	–	(90)
	54 (87)	18 Gy/3	3	–	
	49 (65)	12 Gy/2	3	–	
	29 (37)	18 Gy/3	36	8.1%	(124)
	(39)	(8–12 Gy)/1	36	23%	(60)
	24 (32)	18 Gy/3	6	–	(79)
	28 (35)	12 Gy/2	4	3.1%	(125)
	39 (50)	(9–12) Gy/4	4–6	38%	(73)
	21 (36)	20 Gy/4	24	9.7%	(100)
	35 (54)	6 Gy/1; 8 Gy/2	6	3%	(126)
		4 Gy/1; 6 Gy/2	6	44%	
		18 Gy/3	6	0	
		16 Gy/1	6	0	
	12 (17)	15 Gy/3	24	–	(78)
	147 (147)	12 Gy/4	0.5–1	3.4%	(74)
LDR	31 (46)	(12–18) Gy/1	4–6	30.4%	(73)

Interstitial brachytherapy is followed by irradiation of the keloid by catheter delivery of the radiation source (inserted at a depth of approximately 5 mm) to avoid damage to the surrounding normal tissue (76). Interstitial brachytherapy can be divided into low and high-dose-rate modalities based on the difference in dose (Table 3). Studies have shown that both LDR and HDR brachytherapy is considered safe and effective (77). Since low-dose-rate brachytherapy patients need to be treated in a lead chamber for 20–72 h, although it can effectively treat keloids, it has been replaced by high-dose-rate brachytherapy due to inconvenience and poor patient compliance. HDR brachytherapy is mostly performed within 24 h after surgery, with short irradiation time (less than 10 min) and high patient compliance, and is suitable for outpatient. Surgery combined with HDR brachytherapy significantly reduces the recurrence of keloids with a high patient satisfaction rate (at 86.9%) (74). For patients who failed surgical or postoperative ERBT, adjuvant HDR brachytherapy had a local control rate of 88% (78). In a study of 24 patients with a total of 32 keloids, patients underwent keloid surgery with three HDR brachytherapy at a dose of 6 Gy and a treatment depth of 5 mm, with a mean follow-up of 29.4 months and a local control rate of 94%(79). Tresoldi et al. (80) reported a study of intraoperative radiation therapy with a postoperative control rate of 90.5%, providing a new perspective on early brachytherapy. Although HDR brachytherapy is highly indicated, it has the disadvantage of catheter dislodgement, which has prompted the emergence of surface brachytherapy.

Surface brachytherapy is a way to treat superficial lesions by uniformly adsorbing radionuclides (32P, 90Sr, or 90Y, etc.) onto filter paper or silver foil, making a special dressing radiator according to the shape and size of the lesion, and applying it close to the surface of the lesion (81). Wagner et al. (82) treated 166 keloids in 139 patients with surgical excision and an adjuvant 90Sr-90Y integrated applicator at a median total dose of 14 (7.5–28.5) Gy. The results showed that the recurrence rate was 20.5% and varied by the site of disease, with the lowest recurrence rate in the face and neck (2%) and the highest recurrence rate in the chest (49%). Surface brachytherapy has been widely used in the treatment of keloids because of its non-invasiveness, ease of operation, low adverse effects, low price, and variability in the shape of the applicator. And the long-term follow-up results showed no secondary malignancies in the irradiated area (82).

HDR interstitial brachytherapy is the primary modality of brachytherapy. After comparing multiple publications on treatment regimen selection, their treatment regimens ranged from 2 to 3 sessions delivering 12–18 Gy. After adjusting for differences in gender, skin type, and keloid characteristics, no differences in recurrence rates emerged (76, 79, 83). HDR resulted in better symptom relief and was convenient and less costly to treat (73). When the keloid is uneven and tends to be curved, brachytherapy is often chosen.

## Heavy ion radiotherapy

In recent years, heavy ion radiotherapy has been applied in the clinical treatment of refractory and radiation-insensitive tumors, but its application in the postoperative treatment of keloids has been rarely reported. Chen et al. (84) first performed carbon ion therapy after keloid surgery, and 16 patients received a total dose of 16 Gy in 8 sessions, with a mean follow-up of 29.7 months, showing a cure rate of 95%, and none of the patients had any complications. Heavy ion radiotherapy can achieve ideal dose distribution and biological effects by giving precise doses to the tumor site, disrupting the DNA double-strand of tumor cells, with significant cell-killing effects, and significantly reducing damage to normal tissues and endangered organs, with high efficacy and safety. Thus, heavy ion radiotherapy may be a potential clinical treatment modality for keloids.

## Factors influencing the efficacy of radiotherapy

The multiplication time of normal fibroblasts is 43.5 h. The multiplication time of keloid cells is shortened to 29.5 h, and the rapidly proliferating cells are more sensitive to radiation (85); therefore, adjuvant radiotherapy should be performed as early as possible to obtain better clinical results, and the results of clinical studies show that radiotherapy performed within 24 h after surgery has the best effect (58).

In addition to the choice of radiotherapy timing, the choice of dose and mode is also crucial to the final treatment effect. Too high a dose may damage the normal tissues around the target area and cause serious adverse effects; too low a dose may not achieve the best therapeutic effect. Fractionation therapy allows normal skin cells to recover while keloid cells transition from the radiation-resistant stage to the radiation-sensitive stage. Currently, two segmentation treatment modalities are widely recommended. (1) Multiple splitting mode: a total dose of 17.5–20.0 Gy done in 4 or 5 sessions, each 1 day apart; (2) few splitting mode: a total dose of 18 Gy done in 2 sessions, each 1 week apart. The former is the traditional postoperative radiotherapy modality for keloids, and its safety and efficacy have been proven; however, some investigators believe that the latter is more effective and can be tried for the prevention of postoperative recurrence in refractory and high-recurrence keloids (86). At present, the appropriate radiation dose for the prevention of postoperative keloid recurrence is inconclusive and still needs to be selected by weighing the effectiveness of the treatment against the potential risk of adverse effects.

## Safety and adverse effects of radiotherapy

The potential of radiotherapy as a treatment modality to induce malignancy has been of wide concern. In the X-ray era before 1990, X-ray therapy was reported to have a zero carcinogenic rate (87), which provided supporting evidence for its clinical application. In a study by Preston et al. (88), the probability of developing and dying from skin cancer was 1/7500 in a population aged 18–64 years with whole-body exposure to an irradiation dose of 1 Gy. Based on this study, the postoperative radiotherapy regimen for earlobe keloids reported by Ogawa et al. (89) would expose approximately 0.05% of the patient’s whole body to an 8 Gy dose of irradiation, with an expected morbidity and mortality rate of only 1/2000000 from secondary carcinoma in nearly 4000 patients treated with this regimen. Therefore, the safety of radiotherapy is high.

In the treatment of keloids, radiotherapy may also cause associated complications, which can be divided into early and late complications depending on the time of appearance of symptoms. Early complications include erythema, desquamation, and transient hyperpigmentation, and such adverse effects are seen in all patients within 7–10 days after treatment; (9) late complications are symptoms observed several weeks after treatment, and include permanent hyperpigmentation, capillary dilatation, subcutaneous fibrosis, and chronic ulceration, and the risk of complications after radiotherapy is positively correlated with the irradiation dose (90). In clinical practice, the risk of complications is generally reduced by reducing the fractionated dose while keeping the total dose constant and by lengthening the interval.

## Conclusion and research prospects

Electron beam radiotherapy is high-energy electrons by accelerator-generated. The dose of electrons in the tissue decays rapidly, making electron beam suitable for radiotherapy of skin surface tumors, which minimizes damage to deeper tissues. It can deliver different energies, typically 4–20 MV, and higher energy beams can treat deeper tissue and will have a higher surface dose (91). Although electron beam radiotherapy has advantages in the treatment of superficial lesions, it has complex difficulties, including complicated dose calculations, high costs, and variability of treatment protocols for small lesions (92). Therefore its treatment protocol design in irregular structural lesions is more difficult and prone to computational errors. Because the electron beam used for surface radiotherapy requires high energy maintenance and is generated exclusively by the accelerator, treatment is more expensive.

X-ray differs from electron beam in that it delivers a beam of photons rather than a beam of electrons. The photon beam interacts with the tissue and thus undergoes an ionization reaction to produce secondary electrons. Typically, a lower energy photon beam is produced by an x-ray tube operating in the kilovolt range (89). Compared to electron beam radiotherapy, X-ray has a simpler dose calculation and it is easier to obtain the maximum skin surface dose. The lower energy beam avoids damage to deep structures and can be done on an outpatient basis without the need for hospitalization (93). Since X-ray for the treatment of keloids can be produced by x-ray tubes, the cost is greatly reduced compared to electron beam radiotherapy. However, the low energy of X-ray results in a faster tissue dose and increases the deposition dose in the bone (94). There is less literature on X-ray for keloid treatment (Table 1), and its role in the treatment of skin lesions is not as clear as that of electron beam radiotherapy (95).

In contrast to the two forms of ERBT, brachytherapy is divided into interstitial and surface brachytherapy, depending on the placement of the applicator. Interstitial brachytherapy is further divided into HDR and LDR based on the dose. LDR brachytherapy requires patients to be treated in a lead room for a longer period, usually 20–72 h (96). In contrast, HDR brachytherapy can be performed in an outpatient department because of its short treatment time, proven better patient tolerability, and lower cost (97). And with better symptom relief after HDR treatment (91.7% after HDR vs. 67.9% after LDR, *p* = 0.03), the use of interstitial brachytherapy in keloid treatment is currently limited to the HDR modality (Table 3) (98). Compared to interstitial brachytherapy, surface brachytherapy reduces the risk of catheter displacement and wound dehiscence, especially in the context of extended treatment periods. Brachytherapy treatment requires multiple treatments in a short period to minimize the risk of wound dehiscence and infection (99). The indications for the application of brachytherapy are similar to the previous indications for ERBT, but because it can be tailored to the shape and size of the keloid and whether it is curved, it can be better adapted to different cases (100). Compared to ERBT, brachytherapy is recommended not to involve more surrounding healthy tissue and requires a lower dose of radiation to achieve the same treatment effect (101). Therefore, despite the similar recurrence rates of external and brachytherapy, brachytherapy is gradually increasing in clinical use due to the convenience and lower cost of treatment leading to high patient compliance.

Heavy ion radiotherapy is a new application in keloid. There are very few literature reports on its efficacy and safety, but based on the only literature available, it has a low recurrence rate and complication rate. As research on keloid treatment continues to advance and clinical translation continues, it is likely to become a new form of radiotherapy for keloid treatment in the future.

X-ray are less commonly used in clinical practice because of the paucity of research literature and the lack of multicenter data on recurrence rates and complications. Currently, HDR brachytherapy and electron beam radiotherapy represent the two main radiation modalities for keloid treatment. For curved, irregularly shaped lesions brachytherapy can be used, while for larger areas, thicker tissues, and recurrence-prone sites electron beam radiotherapy should be combined. Although the differences in efficacy between the different radiotherapy modalities were not statistically significant, they each have their advantages and disadvantages (102), as shown in Table 4.

**TABLE 4:** Advantages and disadvantages of adjuvant radiation therapy.

Category	Advantage	Defect
X-ray	Short time, low dose, and fewer acute skin complications	Large damage to the surrounding tissue, Uneven dose delivery
Electron beam	Large surface areas homogeneous dose delivery, less damage to the surrounding tissue	Complex dose calculation, unable to treat the non-plane
Interstitial radiotherapy	High patient compliance, short time	Intralesional catheter insertion, high dose rates
Surface brachytherapy	No trauma, low price, strong plasticity	Easy to fall off
Heavy ion	High effectiveness and safety	Less literature, no clear plan

The review of the literature shows that postoperative adjuvant radiation therapy remains one of the most effective treatments for keloids, with electron beam radiation therapy and HDR brachytherapy being the two main radiation modalities used to adjuvantly treat keloids and provide effective lesion control. Most studies have shown that HDR brachytherapy has better dose coverage and that adjuvant radiation therapy should be administered early (within 24 h), in fewer fractions (usually 4), and at a higher dose (20 Gy) to reduce recurrence rates. Currently, there are numerous studies on postoperative adjuvant radiotherapy for keloids, but no uniform treatment standard has been developed, and future studies should be conducted for the standardized treatment of keloids. Heavy ion therapy may be a potential treatment modality with good future development in the treatment of keloids due to its unique superiority.

## Author contributions

WW: conceptualization, data curation, formal analysis, and writing—original draft. JZ: data curation. CZ: visualization. WZ: investigation. MJ: tables organization. YS: project administration, resources, and supervision. All authors read and approved the final version of the submitted manuscript.
